# Supplementary figures and images for: WilsonGenAI a deep learning approach to classify pathogenic variants in Wilson Disease
Source: PLoS One. 2024 May 17;19(5):e0303787. doi: 10.1371/journal.pone.0303787 (PMC11101024; doi:10.1371/journal.pone.0303787)

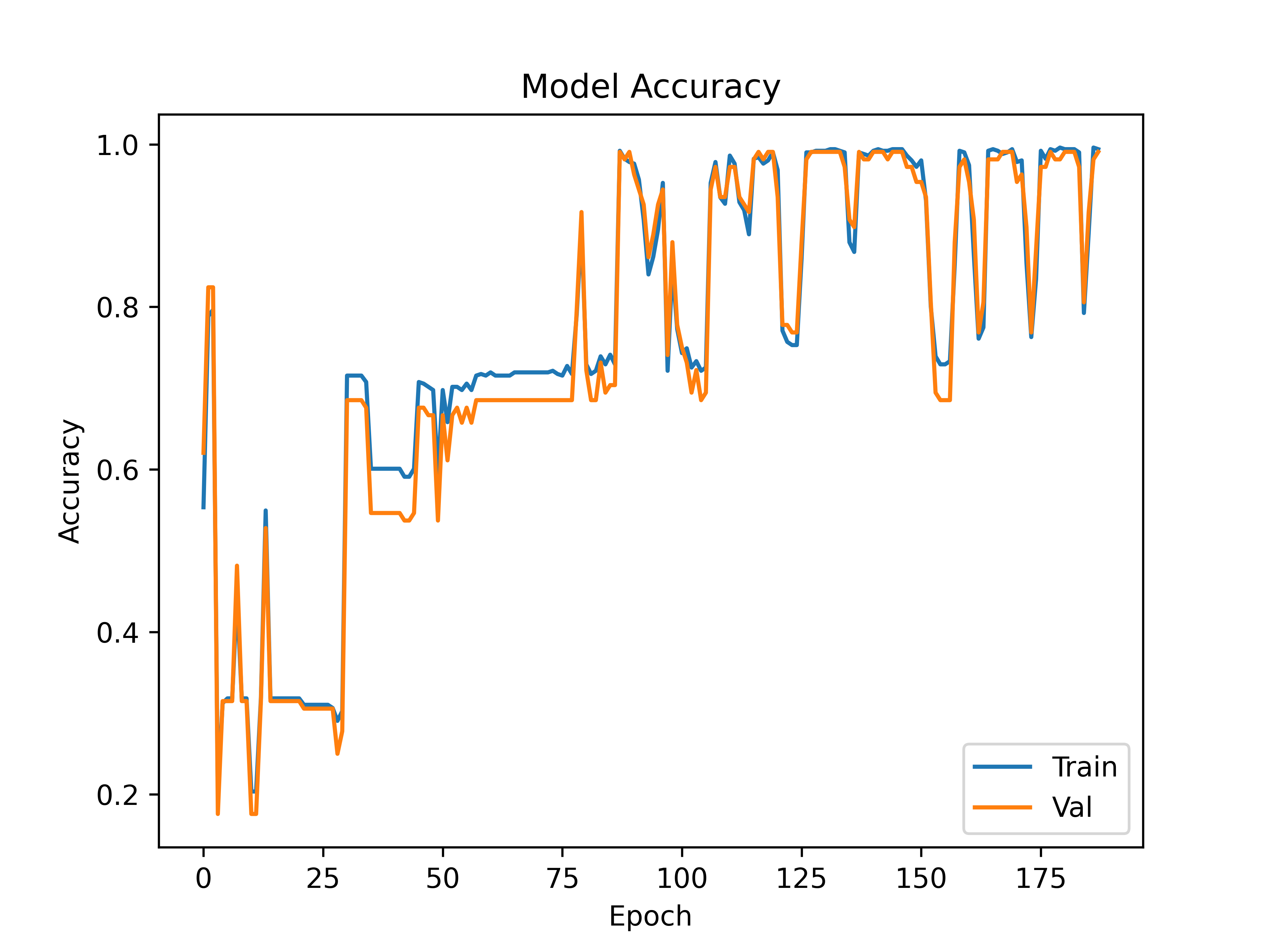

Supplement: S1 Fig — (TIF) [file pone.0303787.s001.tif]

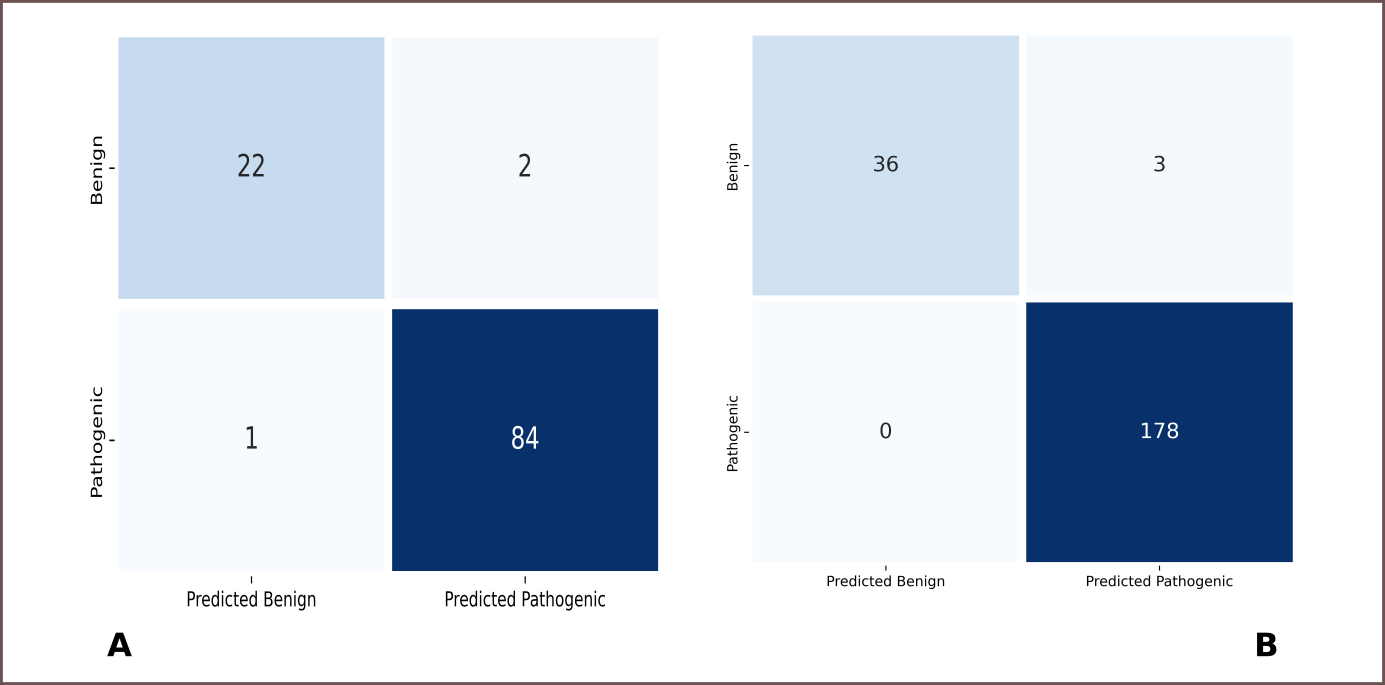

Supplement: S2 Fig — Fig A represents TabNet while B represents XGBoost. (TIF) [file pone.0303787.s002.tif]

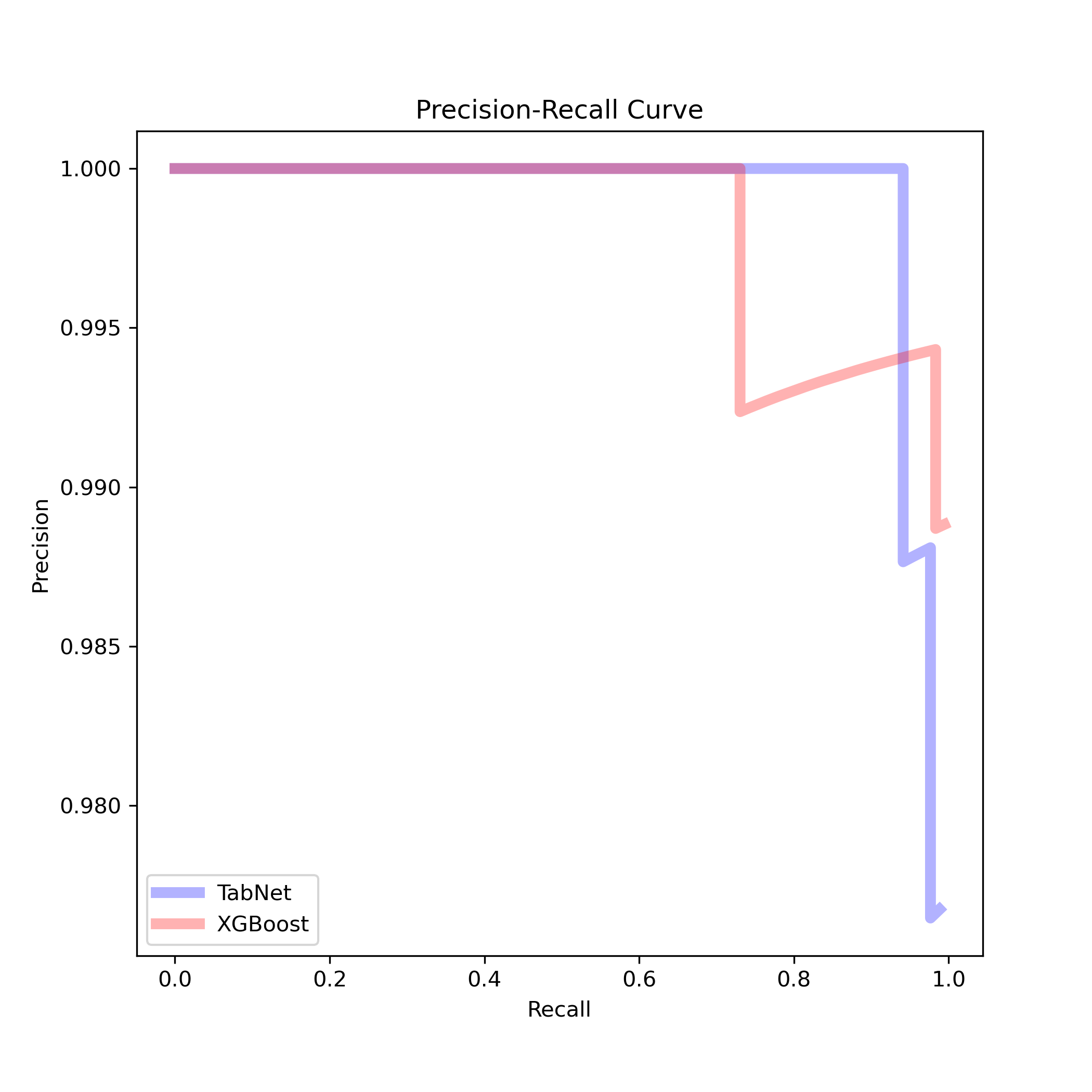

Supplement: S3 Fig — (TIF) [file pone.0303787.s003.tif]

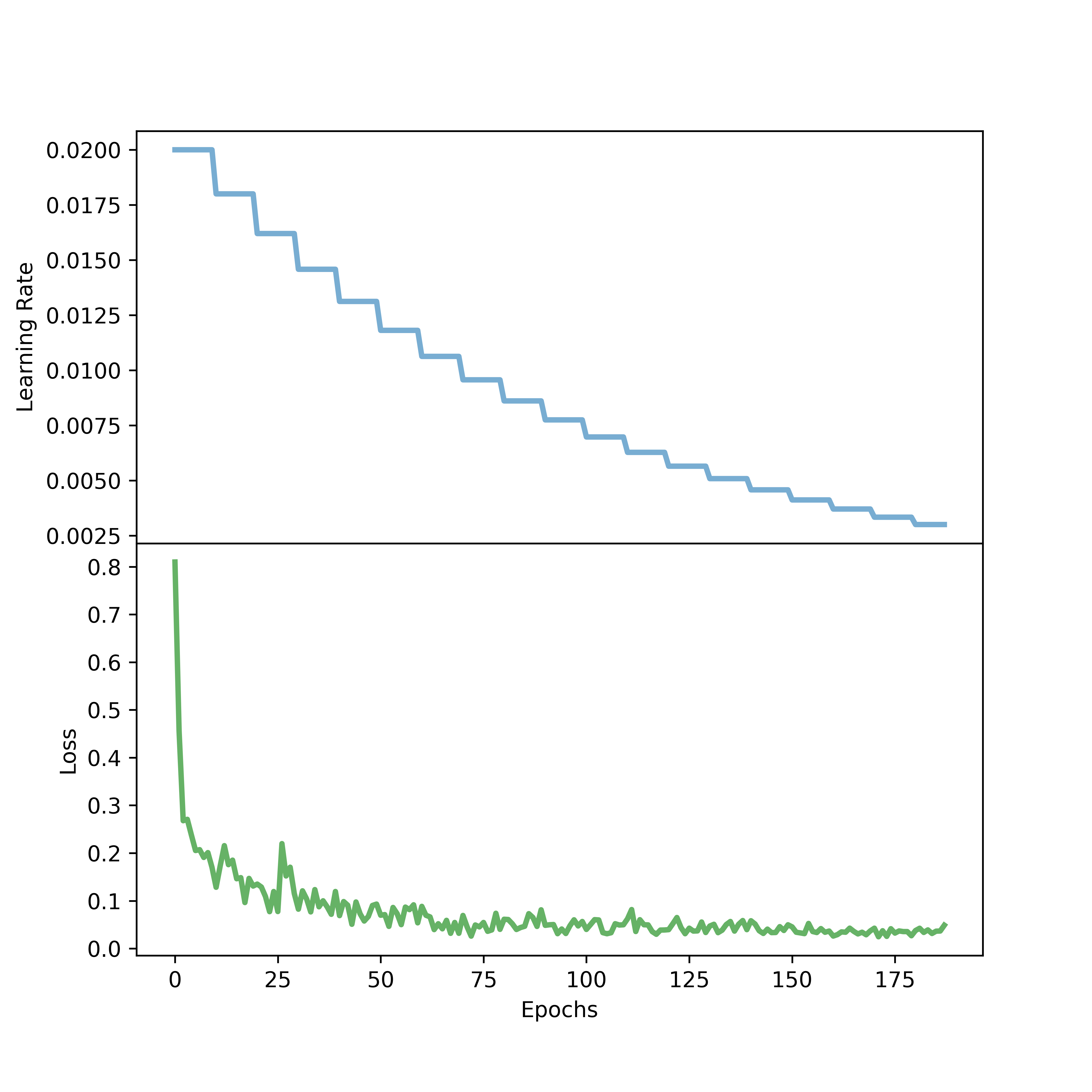

Supplement: S4 Fig — (TIF) [file pone.0303787.s004.tif]

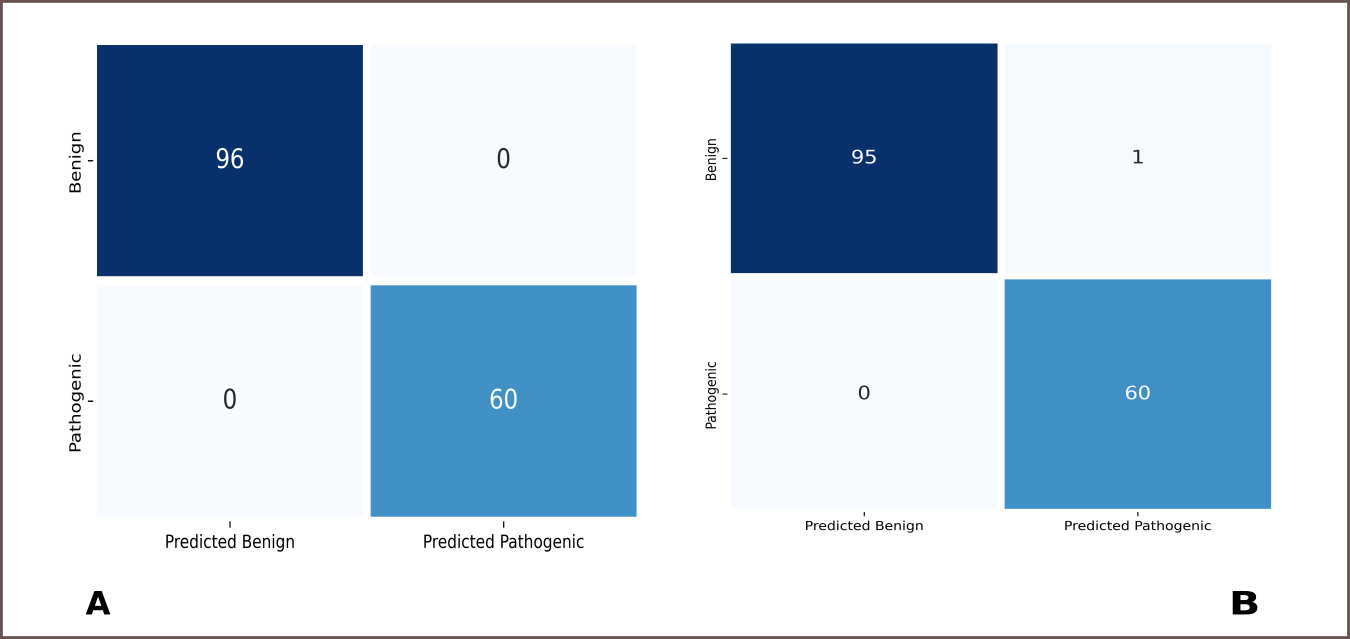

Supplement: S5 Fig — Fig A represents TabNet while B represents XGBoost. (TIF) [file pone.0303787.s005.tif]

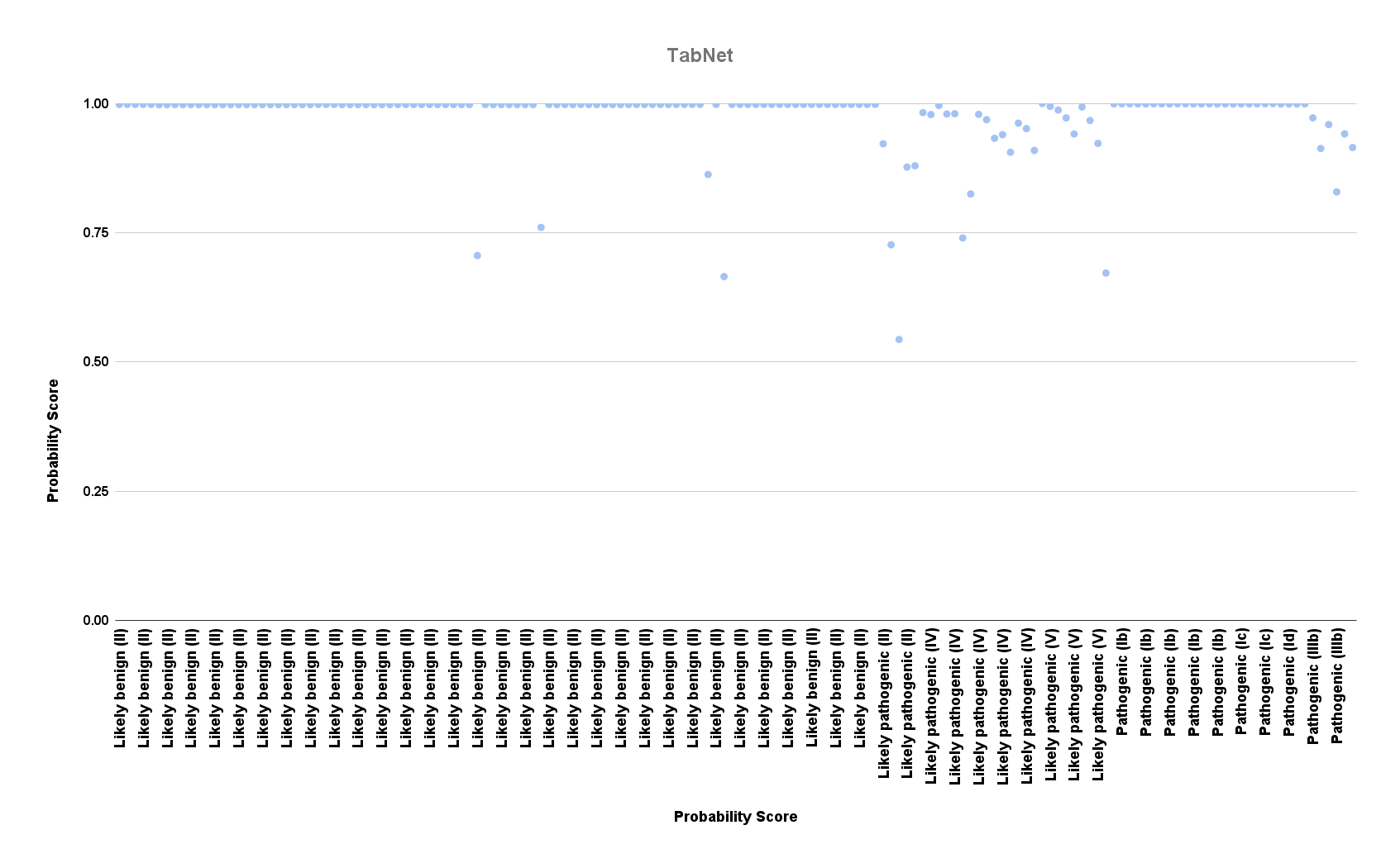

Supplement: S6 Fig — (TIF) [file pone.0303787.s006.tif]

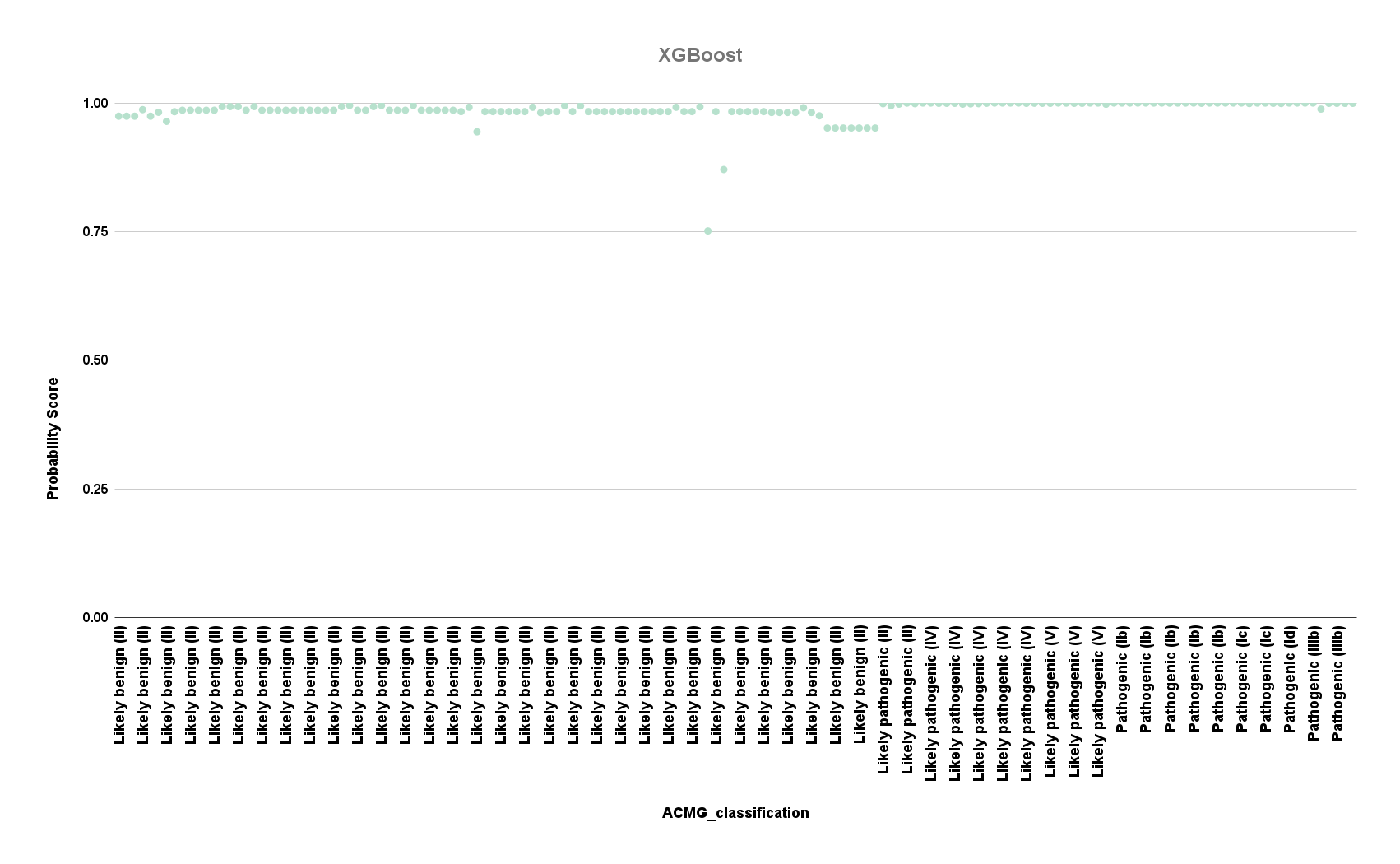

Supplement: S7 Fig — (TIF) [file pone.0303787.s007.tif]

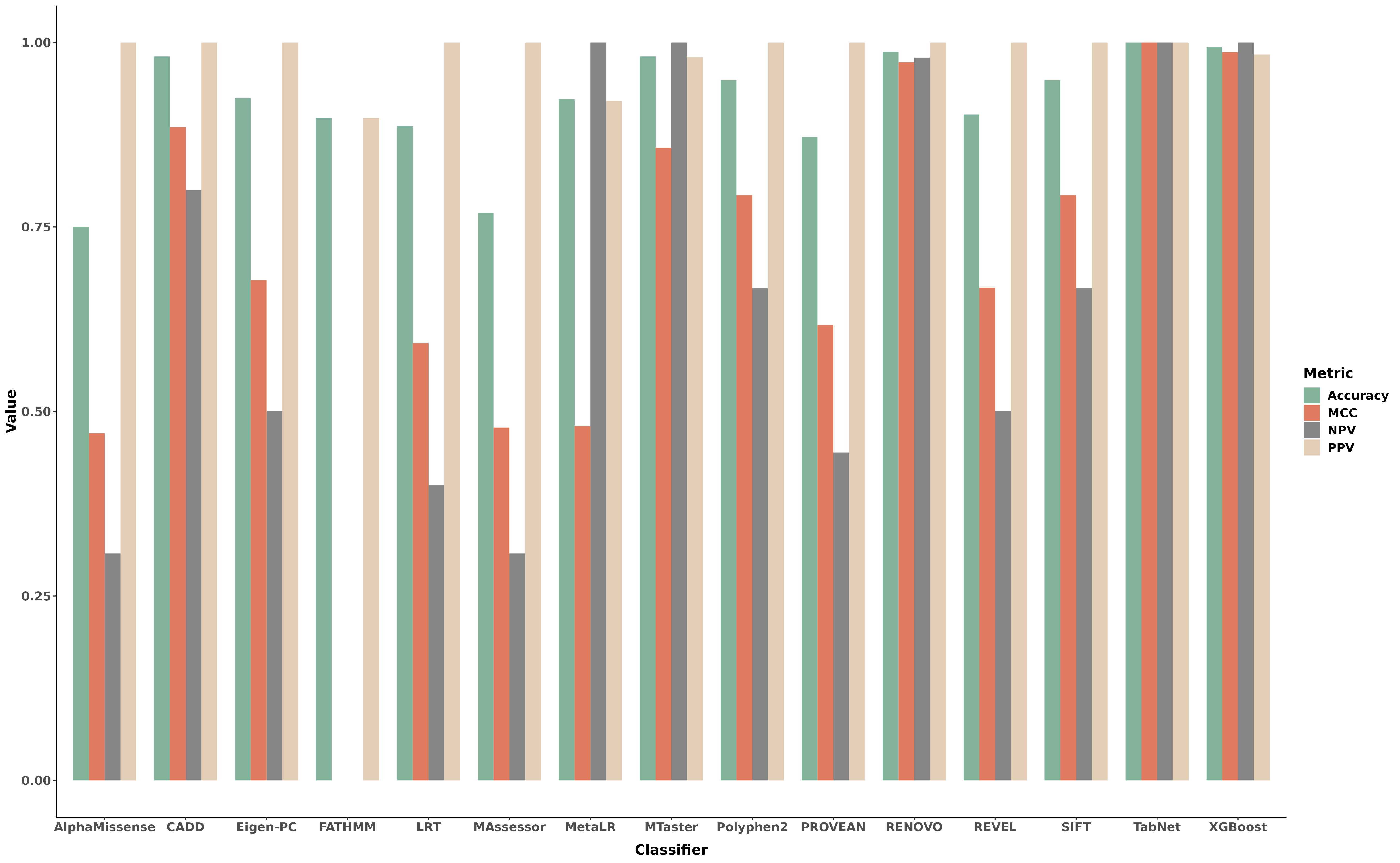

Supplement: S8 Fig — Abbreviations: MAssessor—MutationAssessor; MTaster—MutationTaster. (TIF) [file pone.0303787.s008.tif]

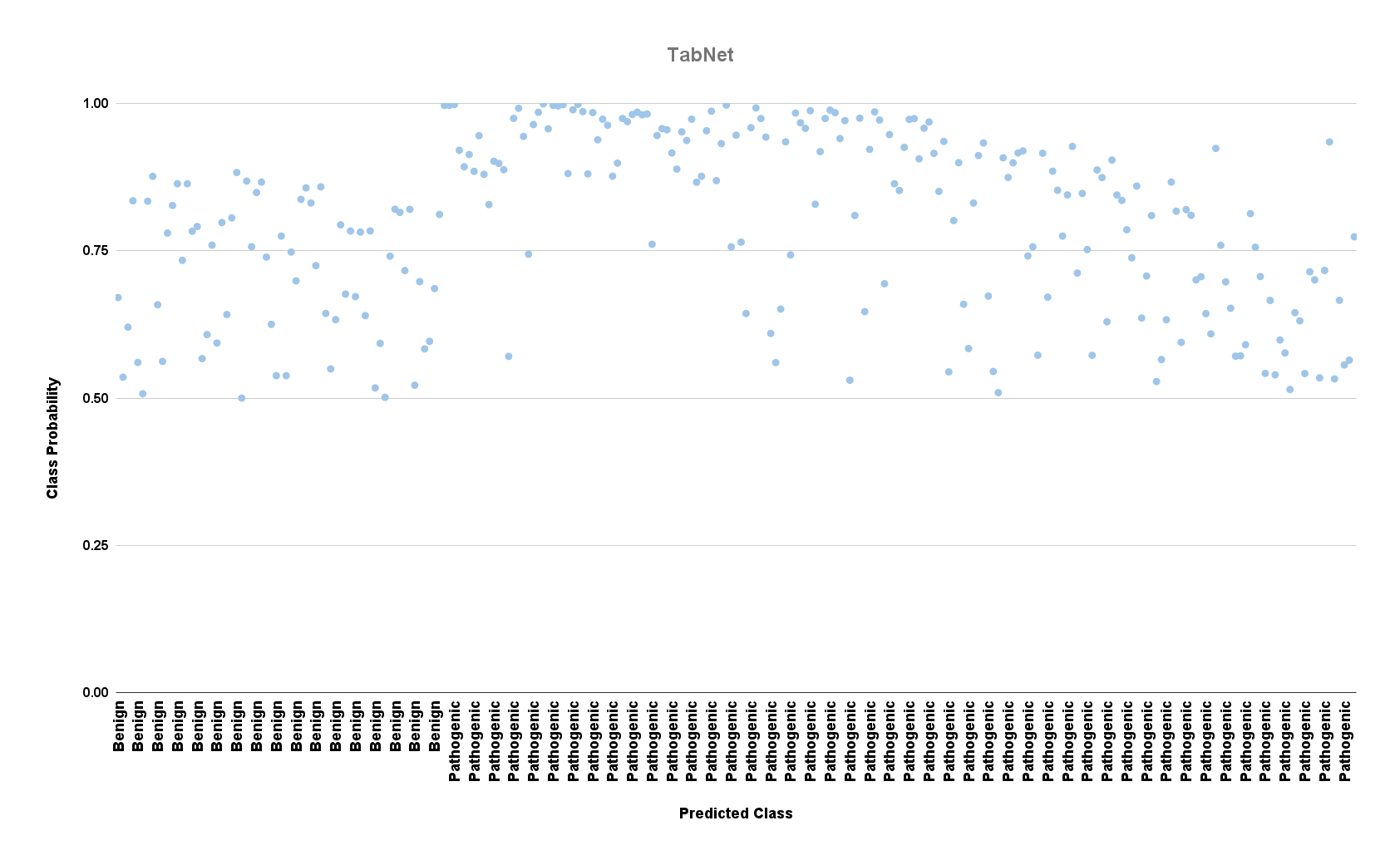

Supplement: S9 Fig — (TIF) [file pone.0303787.s009.tif]

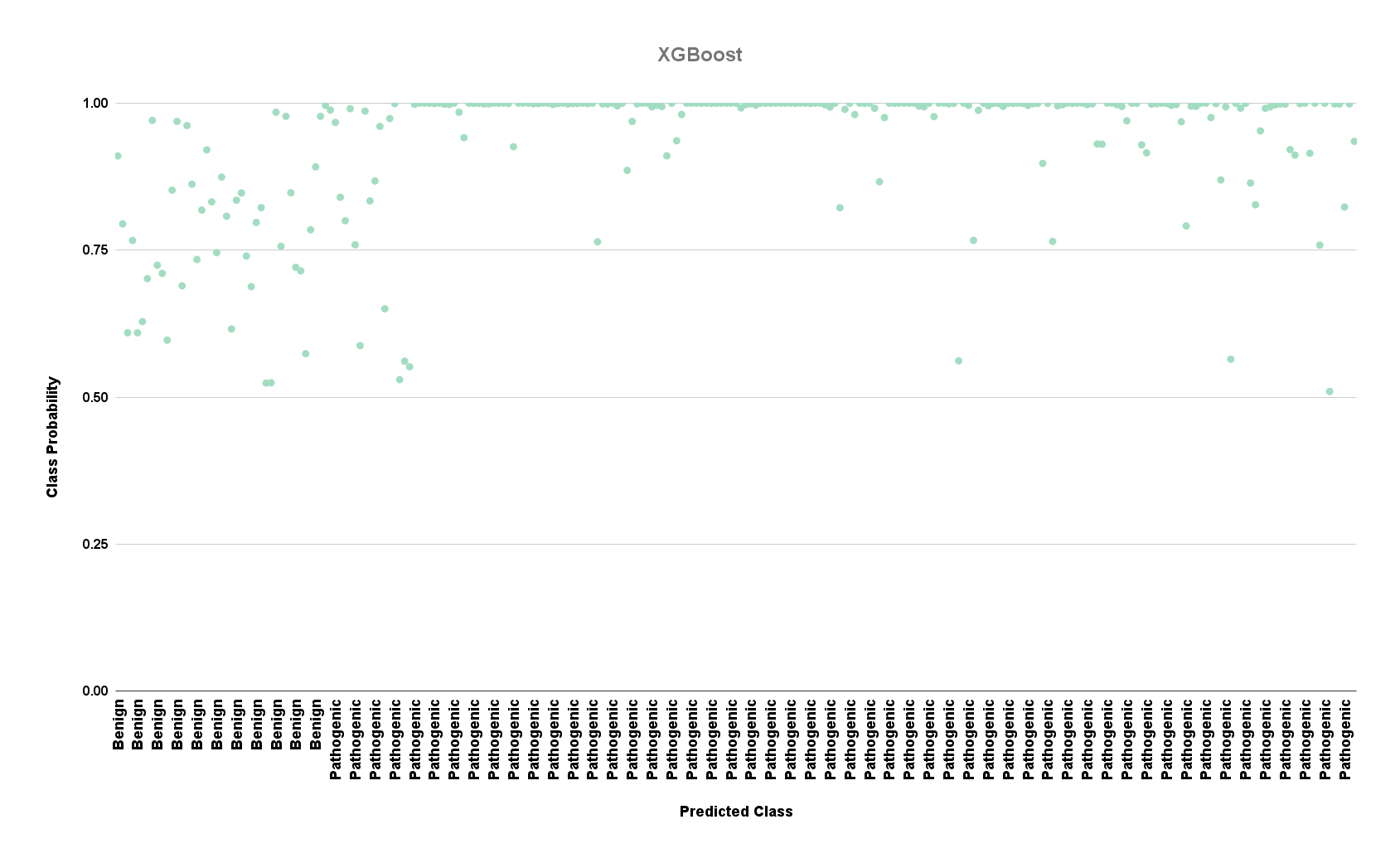

Supplement: S10 Fig — (TIF) [file pone.0303787.s010.tif]

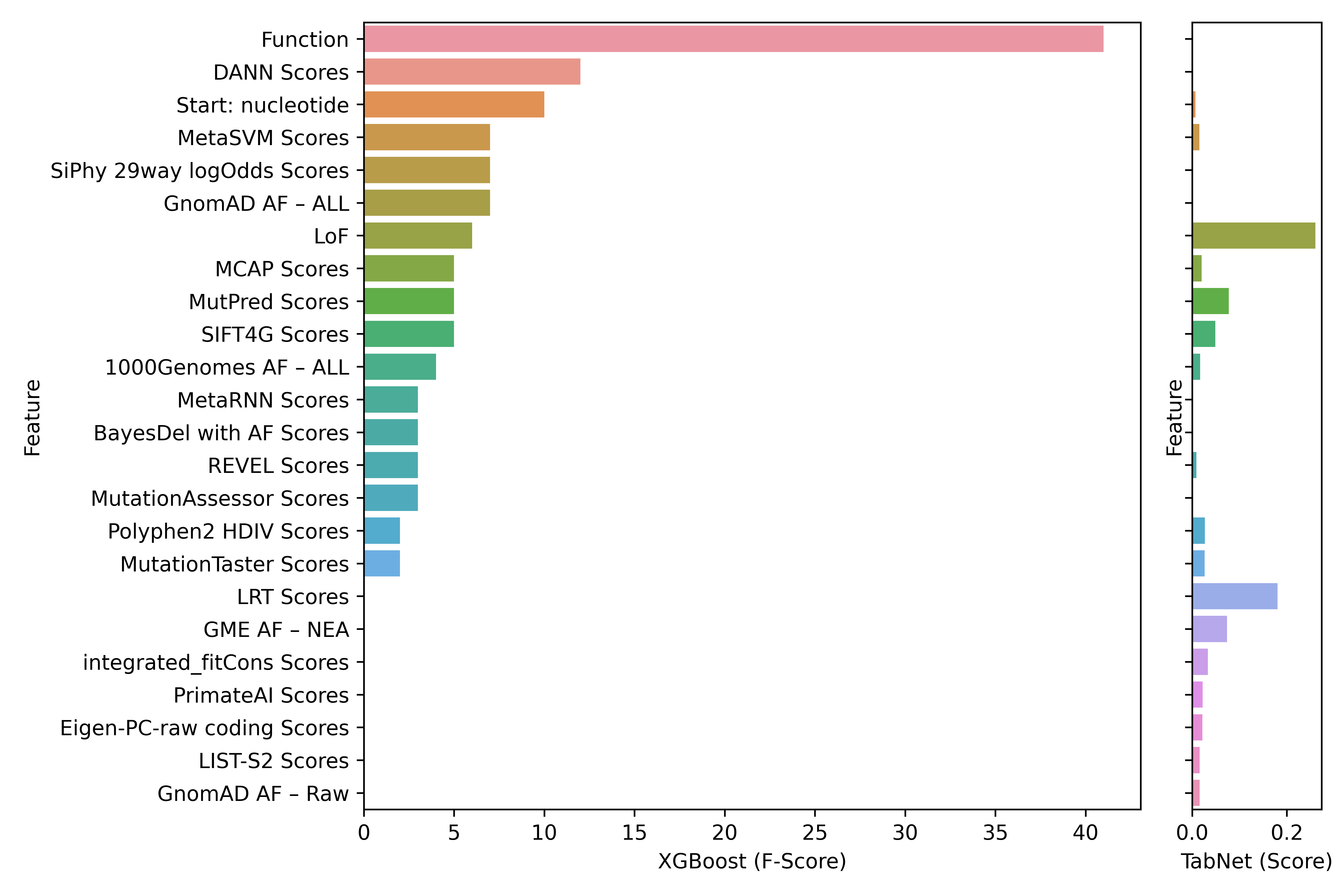

Supplement: S11 Fig — The x-axis for XGBoost plots F-score, while that of TabNet plots scores for each feature. (TIF) [file pone.0303787.s011.tif]

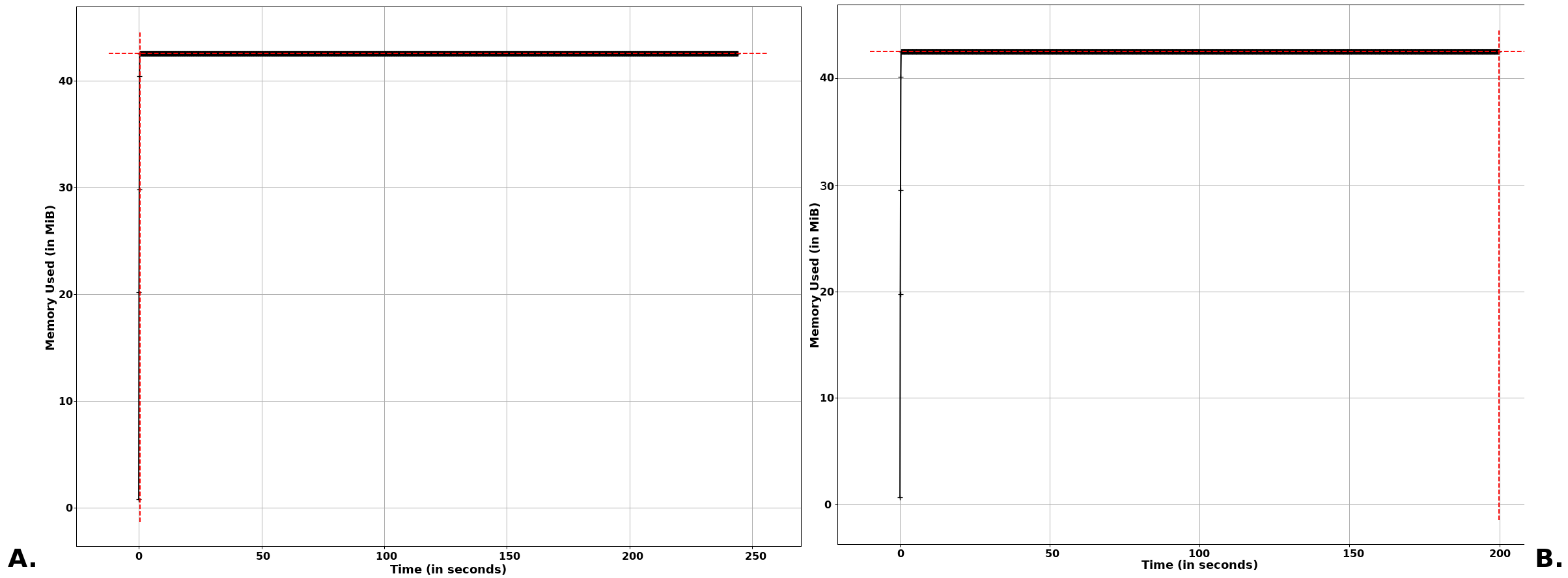

Supplement: S12 Fig — Plot depicting the complete time taken to process a VCF file into suitable input, and then train a model was plotted for (A) TabNet and (B) XGBoost respectively. (TIF) [file pone.0303787.s012.tif]
