# Supplementary material for: WilsonGenAI a deep learning approach to classify pathogenic variants in Wilson Disease
Source: PLoS One. 2024 May 17;19(5):e0303787. doi: 10.1371/journal.pone.0303787 (PMC11101024; doi:10.1371/journal.pone.0303787)
Supplement: Attachment — Submitted filename: ResponsetoReviewers.docx [file pone.0303787.s017.docx]

**A point-by-point response to reviewers**

Reviewer #1: This paper presents genetic variant classification using machine learning techniques, specifically TabNet and XGBoost, to classify ATP7B gene variants associated with Wilson's Disease. The study's strength lies in its robust training and validation on a high-confidence dataset and its practical application, as evidenced by successful independent verification and potential utility in clinical and research settings. I have several comments that are need to be addressed.

Major:

**(Q1) Why were TabNet and XGBoost chosen as the primary models for this analysis over other deep learning or machine learning models? What specific advantages do they offer for this type of data and problem? Please provide the comparison with other relevant deep learning methods.**

**Response:** We appreciate the inquiry from the reviewer. In our initial exploration of model selection, we conducted a comprehensive analysis using the Weka suite (Witten et al. 2011). The dataset under consideration at that time comprised 725 variants, split into 70% training and 30% testing datasets. The table below illustrates the train and test accuracies achieved by different algorithms:

| **Model** | **Train Accuracy** | **Test Accuracy** |
| --- | --- | --- |
| **RandomForest** | 97.925 | 98.611 |
| **J48** | 97.41 | 98.61 |
| **SMO** | 96.89 | 97.92 |
| **NaiveBayes** | 83.59 | 76.39 |

Consistent with conventional wisdom, tree ensemble models demonstrated superior performance on tabular data. Notably, RandomForest and J48 outperformed other algorithms. Therefore, we opted for the XGBoost classifier, a widely-used gradient-boosted decision tree, known for its efficiency in handling tabular datasets. XGBoost offers advantages such as faster execution, robust performance with missing data, and effective handling of class-imbalanced datasets. Its built-in regularization helps mitigate overfitting, a concern associated with models like RandomForest.

Furthermore, recognizing the specialized nature of tabular data, we incorporated the TabNet deep learning model into our analysis. TabNet, designed specifically for tabulated datasets, leverages a transformer architecture to emulate the learning of decision trees. This design enables TabNet to rapidly discern intricate data patterns. While XGBoost excels in certain scenarios, TabNet has demonstrated superiority over tree methods for specific tabular datasets.

Given the variability in the comparative performance of these models depending on the dataset, we decided to include results from both XGBoost and TabNet for a comprehensive evaluation. This dual-model approach allows for a more nuanced understanding and robust assessment of the predictions made by each model.

**(Q2) The authors mentioned that TabNet uses sequential attention for feature selection, which is instance-wise. How does this impact the generalizability of the model across different datasets or variants? Is there a risk of overfitting to specific features in the training dataset?**

**Response:** We appreciate the reviewer's insightful consideration of the generalizability of TabNet across diverse datasets. While TabNet incorporates features like prior scales to mitigate overfitting, predicting the exact extent of model generalization remains challenging, particularly in the absence of ample accurately classified variant datasets. Our training utilized the most extensive dataset of Wilson's Disease variants reported in literature, encompassing nine large datasets with ACMG classifications. The model consistently demonstrated high classification accuracy across both classes, as assessed by Matthews Correlation Coefficient (MCC). This performance instills confidence in its potential to perform well on other real-world datasets. Regrettably, we were unable to conduct additional testing due to the scarcity of available ACMG-classified or functionally validated variant datasets. Despite this limitation, our rigorous training on a diverse and comprehensive dataset enhances our confidence in the model's ability to generalize to different datasets and variants. Future investigations and validations with additional variant datasets would certainly contribute to a more comprehensive understanding of TabNet's generalizability across a broader spectrum of genetic variations.

**(Q3) The authors note that XGBoost is effective in handling sparse data. However, it is not clear that on how this capability was specifically advantageous in current study, given the characteristics of used dataset?**

**Response:** We appreciate the reviewer's observation, and would like to clarify the specific advantage of XGBoost's capability to handle sparse data in our study. Real-world datasets often exhibit missing values, posing a challenge for deep learning models. In our dataset, certain features, such as pathogenicity and conservation scores, had missing values due to the inherent characteristics of their respective prediction algorithms. To address this issue with TabNet, we had to perform imputation by substituting missing data with a constant value far outside the scale of all scores. This substitution aimed to avoid introducing unintended bias.

Contrastingly, XGBoost demonstrated an inherent advantage in handling sparse data. Unlike TabNet, XGBoost required no data substitution for missing values, resulting in a more streamlined preprocessing step. Moreover, XGBoost's Sparsity-aware Split Finding algorithm automatically determines optimal splits for data points with missing values, contributing to an improved overall performance. This capability proved advantageous in our study, in terms of streamlining the preprocessing phase and enhancing the model's efficiency in handling sparse data patterns.

**(Q4) For the models, the authors have set specific hyperparameters. The manuscript need more details about how were these parameters chosen.**

**Response:** We appreciate the reviewer's request for more details on the hyperparameter selection process. In the TabNet model, we explored different values for the mask_type parameter during experimentation. The "entmax" setting demonstrated superior overall prediction accuracy compared to the default "sparsemax," leading us to choose it for model training. The “patience” parameter, governing the number of epochs to await improvement before terminating a training run, was set at 100, with a maximum of 1000 epochs allowed. Various dataset splits were tested, including 70% train and 30% test, as well as 80% train and 20% test, to ensure robust testing.

For the XGBoost model, hyperparameters were carefully selected and evaluated using a 5-fold cross-validation approach. A randomized search on hyperparameters was conducted using RandomizedSearchCV with 5-fold cross-validation. To address class imbalance, the scale_pos_weight parameter was determined by dividing the number of majority class entries by the number of minority class entries. Model performance was assessed using the mean cross_val_score function with a 10-fold cross-validation. Multiple models, with and without the determined hyperparameters (including scale_pos_weight), were tested using accuracy, AUC, and MCC metrics. Additionally, various train/test splits were explored to identify the best-performing model.

These details have been incorporated into the revised manuscript to provide a comprehensive understanding of the hyperparameter selection process for both the TabNet and XGBoost models.

**(Q5) The authors adjusted the scale_pos_weight in XGBoost for class imbalance. How significant was the class imbalance in used dataset, and how did this adjustment impact the model's performance, especially in terms of precision and recall?**

**Response:** Our train set had 577 pathogenic/likely pathogenic and 146 benign/likely benign variants. Given this imbalance, we adjusted the scale_pos_weight parameter to the recommended value of 3.95. To evaluate the impact of this adjustment, we employed the Matthews Correlation Coefficient (MCC) metric, which comprehensively considers all components of the confusion matrix, namely true positives (TP), true negatives (TN), false positives (FP), and false negatives (FN). It thus enabled us to determine if the classifier was doing well on both positive and negative classes. This was pertinent due to the potential clinical implications associated with misclassifying benign variants as pathogenic.

Comparing models with and without the adjusted scale_pos_weight, we observed an improvement in performance with the weighted model achieving an MCC of 0.95 compared to 0.90 without the weights. While precision remained consistent at 0.98 for both models, the weighted model exhibited an enhanced recall of 1.00 as opposed to 0.98 without the adjustment. Moreover, the F1-score demonstrated improvement with the weighted model, reaching 0.99 compared to 0.98 without the adjustment.

It is noteworthy that our pursuit of optimal hyperparameter configurations involved experimenting with various combinations. Throughout this process, models consistently performed better when the scale_pos_weight parameter was appropriately adjusted. This underscores the significance of addressing class imbalance, as reflected in the superior performance and robustness of models that incorporated the weighted approach.

**(Q6) TabNet stopped training at the 187th epoch out of a possible 1000. Was this due to an early stopping criterion based on validation accuracy? A big epoch size does not necessarily increase the accuracy of the model. How was the risk of overfitting addressed given the excessive epoch size (>100)?**

**Response:** Indeed, TabNet implemented an early stopping mechanism based on the validation accuracy metric during training. The early stopping criterion was defined by setting patience at 100, meaning that if the accuracy did not improve for 100 consecutive epochs, the training process would halt. Subsequently, TabNet automatically selected the epoch with the best accuracy score for making predictions on the evaluation set.

To mitigate the risk of overfitting, the model width, representing the number of nodes in a layer, was set to 8. Additionally, the parameter n_steps was configured to 3. These decisions aimed to strike a balance between model complexity and generalization capacity, reducing the likelihood of overfitting.

Furthermore, to validate the robustness of the model, its performance was rigorously assessed on an independent validation set, where it was able to correctly classify all variants across both classes. We thus anticipate the model to be able to generalize well on data beyond the training set.

**(Q7) The authors mentioned the top 20 features in feature importance plots for both models. Could the authors provide insights into what these features represent and how they contribute to the pathogenicity classification? How interpretable are these models in terms of understanding the biological significance of these features?**

**Response:** Certainly, the feature importance plots for both models shed light on the factors influencing pathogenicity classification, derived from a comprehensive training set of 73 attributes. These attributes include variant positional information, global population prevalence, pathogenicity prediction scores from various tools, and evolutionary conservation scores.

The top features identified by both models highlight critical determinants of pathogenicity. Loss of function (LoF) information emerges as a key contributor, emphasizing the significance of genetic lesions that impede normal gene product formation, a hallmark of disease causation. The genomic position of the mutation(Start: nucleotide), could also be important in predicting pathogenic effect. Further, the global prevalence of variants, as indicated by the 1000Genomes allele frequency (1000Genomes AF - ALL), underscores the observation that the number of high frequency disease causing variants is usually small, i.e. most pathogenic variants are rarely prevalent across a population. The remaining features common to both models are pathogenicity scores from seven prediction tools (MetaSVM, MCAP, MutPred, SIF4G, REVEL, PolyPhen2 HDIV, and MutationTaster), reflecting the amalgamation of diverse computational predictions.

The XGBoost model additionally considers exonic function (Function), which describes the nature of the effect the variant has (a stopgain/loss variant for example, would have a larger effect on the protein than a synonymous variant).Allele frequencies from the GnomAD database, representing a larger population dataset, are also considered.It also takes into account conservation scores (Siphy 29way logOdds and MutationAssessor) that dictate how conserved a given site is among mammals, indicating a potentially important location, and thus a potentially more disruptive effect. The model further incorporates pathogenicity scores from DANN, MetaRNN, and BayesDel.

The TabNet model additionally considersvariant prevalence across Gnomad (GnomadAF - Raw) and the Northeast African subset of the Greater Middle East populations (GME AF - NEA). Pathogenicity and conservation scores, including LRT, integrated_fitCons, PrimateAI, Eigen-PC-raw coding, and LIST-S2, enhance the model's ability to capture nuances in variant significance.

Thus both models take a well-rounded approach, and consider different aspects that determine variant pathogenicity, and are thus able to make reliable predictions. Further, the train dataset labels have been determined through ACMG classification that take into account all aspects of relevant biological data including functional and segregational evidence. As such the models capture patterns among the attributes that lead to these classifications.

The table below is a subset of Supplementary Table 1, and offers greater detail on each of the top 20 important features:

| **Feature Name** | **ACMG2015** | **Description** | **Dtype** |
| --- | --- | --- | --- |
| Function | PVS1, BP7 | Exonic function of the variant (e.g.: nonsynonymous SNV, stopgain/loss, frameshift insertion/deletion etc.) | **category** |
| DANN Scores | PP3, BP4 | Deleterious Annotation of genetic variants using Neural Networks. Score range: 0-1. | float64 |
| Start: Nucleotide |  | Genomic location of nucleotide | int64 |
| MetaSVM | PP3, BP4 | A radial SVM model to predict pathogenicity, trained on whole exome variants. Score range: -2 to 3. | float64 |
| Siphy 29way logOdds Scores | PP3, BP4 | SiPhy score based on 29 mammals genomes. The larger the score, the more conserved the site. Score range: 0 to 37.9718. | float64 |
| Gnomad AF - ALL | BA1, BS1, BS2, PM2 | Alt allele Frequency in the GnomAD database | float64 |
| LoF | PVS1, BP7 | Whether a variant is High Confidence LoF | **category** |
| MCAP Scores | PP3, BP4 | Pathogenicity classifier for rare missense variants in the human genome. Score range: 0-1. | float64 |
| MutPred Scores | PP3, BP4 | Automates the inference of molecular mechanisms of disease from amino acid substitutions. Models changes of structural features and functional sites between wild-type and mutant protein sequences | float64 |
| SIFT4G Score | PP3, BP4 | Faster implementation of SIFT for wider range of organisms. Score range: 0-1. | float64 |
| 1000Genomes AF -ALL | BA1, BS1, BS2, PM2 | Allele frequency in the 1000 Genomes database | float64 |
| MetaRNN Scores | PP3, BP4 | Pathogenicity prediction scores for human nonsynonymous SNVs (nsSNVs) and non-frameshift (NF) indels. | float64 |
| BayesDel with AF Scores | PP3, BP4 | Deleteriousness meta-score for coding and non-coding variants, SNVs and small insertion / deletions from database with integrated MaxAF. Score range: -1.29334 to 0.75731. | float64 |
| REVEL Scores | PP3, BP4 | Predicting the pathogenicity of missense variants on the basis of individual tools: MutPred, FATHMM, VEST, PolyPhen, SIFT, PROVEAN, MutationAssessor, MutationTaster, LRT, GERP, SiPhy, phyloP, and phastCons. Score range: 0-1. | float64 |
| MutationAssessor Scores | PP3, BP4 | Predicts the functional impact of amino-acid substitutions in proteins, such as mutations discovered in cancer or missense polymorphisms. The functional impact is assessed based on evolutionary conservation of the affected amino acid in protein homologs. Score range: -5.545 to 5.975. | float64 |
| Polyphen2 HDIV Scores | PP3, BP4 | Polyphen2 prediction based on HumDiv; The PolyPhen-2 score predicts the possible impact of an amino acid substitution on the structure and function of a human protein. Score range: 0-1. | float64 |
| MutationTaster Scores | PP3, BP4 | Evaluates disease-causing potential of sequence alterations. Analyses comprise evolutionary conservation, splice-site changes, loss of protein features and changes that might affect the amount of mRNA. Test results are then evaluated by a naive Bayes classifier2, which predicts the disease potential. Score range: 0-1. | float64 |
| LRT Scores | PP3, BP4 | Likelihood ratio test (LRT) to identify a subset of deleterious mutations that disrupt highly conserved amino acids within protein-coding sequences, which are likely to be unconditionally deleterious. Score range: 0-1. | float64 |
| GME AF - NEA | BA1, BS1, BS2, PM2 | Greater Middle East Variome allele frequency for samples of northeast African ancestry | float64 |
| integreated_fitCons Scores | PP3, BP4 | Fitness consequences of functional annotation. Score range: 0-1 | float64 |
| PrimateAI Scores | PP3, BP4 | Deep residual neural network for classifying the pathogenicity of missense mutations. Trained on a dataset of common missense human variants and six non-human primate species | float64 |
| Eigen-PC-raw coding scores | PP3, BP4 | Scores representing the first principle component of the covariance matrix | float64 |
| LIST-S2 Scores, | PP3, BP4 | Tool offering taxonomy based sorting of deleterious missense mutations across species. | float64 |
| GnomadAF - Raw | BA1, BS1, BS2, PM2 | Alternate allele frequency in samples, before removing low-confidence genotypes | float64 |

**Table 1.** : A subset of Supplementary Table 1, offering greater details on the top 20 important features across both models

**(Q8) The manuscript needs more details on how specificity, negative predictive value (NPV), or area under the precision-recall curve (AUPRC) considered?**

**Response:** We appreciate the reviewer's feedback and have incorporated additional details on specificity, negative predictive value (NPV), and area under the precision-recall curve (AUPRC) in the manuscript. Notably, both models demonstrated specificity and NPV exceeding 0.989. Furthermore, the average precision score (AUPRC) was determined to be 1 for both models, as shown in the figures below. We have added these details to the manuscript as well.

**Fig 1.** : Precision-Recall curve for the XGBoost model.

**Fig 2.** : Precision-Recall curve for the TabNet model.

**(Q9) The test sets' composition (number of benign vs. pathogenic variants) and their source (whether they were balanced or reflective of real-world distributions) are not detailed. How might this affect the models' generalizability to other datasets or real-world scenarios?**

**Response:** In our study, both the test and train datasets have been obtained from WilsonGen, an extensive database comprising over 1878 ATP7B variants meticulously classified in accordance with the ACMG/AMP guidelines. These guidelines represent the gold standard in variant classification, ensuring a high level of accuracy and reliability in the training and evaluation of our models.

WilsonGen stands out as a comprehensive resource, having systematically gathered and classified variants from various sources, including publicly reported literature and nine large databases specifically containing Wilson’s Disease mutations. This meticulous curation makes WilsonGen the most extensive and detailed database of ACMG/AMP classified variants, to the best of our knowledge. It is thus an effective resource designed to enable accurate disease diagnosis and genetic counseling. Given the robust nature of WilsonGen, our models have been trained on a diverse set of variants, providing a representative sample of pathogenic and benign instances. This diversity plays an essential role in capturing the complexity and heterogeneity of genetic variations associated with Wilson's Disease.

We anticipate that the models developed and validated on WilsonGen will exhibit a high degree of generalizability to other datasets and real-world clinical or research scenarios. The extensive and varied nature of the data, coupled with adherence to gold-standard classification guidelines, enhances the models' ability to discern patterns and accurately classify variants in different genetic landscapes. We have elaborated on the robust nature of WilsonGen in te revised manuscript to highlight this point.

**(Q10) The comparison with CADD and other models like RENOVO and MLVar suggests superior performance of your models. However, were these comparisons made under similar conditions (e.g., same datasets, metrics)? How do the models compare in terms of computational efficiency and scalability?**

**Response:** Yes, all model comparisons were conducted on an independent test set comprising of 156 variants, ensuring a fair and consistent evaluation framework. The assessment was based on the same set of metrics, including Accuracy, Positive Predictive Value (PPV), Negative Predictive Value (NPV), and Matthews Correlation Coefficient (MCC). In addition to comparing our models with CADD, we extended the analysis to include 13 other models, namely RENOVO, AlphaMissense, REVEL, SIFT, Polyphen2, Eigen-PC, LRT, MutationTaster, FATHMM, PROVEAN, MetaLR, and MutationAssessor. Our models’ performance exceeds that of others, as detailed in Supplementary Table S3.

The RENOVO model was freely available to implement, and was successfully run to obtain scores. We were unable to install CADD despite repeated attempts on two different systems due to issues with the conda environment provided. The scores generated by the remaining models were either collected through the ANNOVAR tool, or through the repositories made available by the authors.

We were able to run RENOVO on the Ubuntu 18 LTS system used to create and run our models. While RENOVO took approximately 18 minutes to annotate the VCF and generate scores, our pipeline took approximately 1 minute for annotation, and a few seconds by each model for generating predictions on the test dataset of 156 variants. The complete time taken to process a VCF file into suitable input, and then train a model was plotted for each model separately, and has been added below as one graph for each model. They have additionally been added to the manuscript as Supplementary Figure 12.

While the assessment of scalability was limited due to the absence of sufficient blind data, the efficiency and superior performance of our models underscore their potential for practical implementation in real-world scenarios, and their ability to handle large-scale genetic variant classification tasks.

**Fig 1.** : Memory profiling for the TabNet model

**Fig 1.** : Memory profiling for the XGBoost model

**(Q11) When reclassifying variants of uncertain significance, how did the authors validate the accuracy of these reclassifications? Is there a risk of introducing bias or errors in this process, given the uncertain nature of these variants?**

**Response:** ML approaches offer a means of rapidly classifying large numbers of variants, which would otherwise not be possible through functional classification or ACMG approaches, both of which require significant amount of time, and are dependent on expertise for accuracy. While we are attempting to perform the copper assay for validation, it is an ongoing process that is expected to take time. As the copper assay results become available, we will further refine and validate the reclassifications, ensuring a robust and comprehensive evaluation of the ML-based variant classification process.

In the interim, to mitigate the risk of misclassification, we have made the pathogenicity predictions from both models available for consideration. Out of a total of 977 variants, we observed a noteworthy 91.4% concordance in predictions between the two models. The predictions can be found in Supplementary Table 4, where the concordant ones have been highlighted in bold. To provide additional context regarding prediction confidence, we have included the class probabilities for each model alongside the predictions.

**(Q12) The discussion section of the manuscript needs to be significantly expanded. These are few points the authors may consider while revising the discussion section. In discussion the authors should interpret and explain the findings, placing them in the context of the broader field. Begin by summarizing the main findings of the study, highlighting how they address the research questions or hypotheses stated in the introduction. Then, contextualize these results within the existing literature, discussing how these findings align with or differ from previous research and the potential reasons for these similarities or differences. What is the significance and implications of the results, considering both their theoretical and practical applications. Acknowledge the limitations, discussing how they might affect your findings and suggesting areas for future research to address these gaps. This section should bridge the gap between the presented research and the larger scientific community, demonstrating how this work contributes to and advances the field.**

**Response:** We appreciate the valuable input from the reviewer and have thoroughly revised the Discussion section in response to the provided suggestions.

Minor:

**(Q1) It would help readers to introduce Wilson's disease in the introduction section.**

**Response:** We appreciate the reviewer's suggestion, and in response, we have enriched the introduction section of the manuscript to provide a more detailed and informative overview of Wilson's disease. This addition aims to ensure that readers, including those less familiar with the condition, can better understand the context and significance of the study.

**(Q2) The relevance of choosing the ATP7B gene needs to be added in the introduction.**

**Response:** We express gratitude to the reviewer for the insightful feedback. In response, we have incorporated additional details in the introduction section to elucidate the relevance of selecting the ATP7B gene for our study. This addition aims to provide a clearer understanding of the rationale behind focusing on this specific gene and its significance in the context of Wilson's disease.

**(Q3) "Non-exonic variants and VUS were removed from the analysis and this resulted in a variant dataset of 723 unique variants, ..." Explain why. What is VUS? Expand all the abbreviations at the first use.**

**Response:** Exonic variants typically encompass the majority of causative variants in various diseases. In our dataset, non-exonic variant types, including intronic, splicing, upstream, UTR3, and UTR5 variants, collectively constituted only 13% of the data. Consequently, there was insufficient data to effectively train models for each individual type of non-exonic variant.

Variants of Uncertain Significance (VUS) are genetic variations for which a clear classification as either Pathogenic or Benign could not be determined through ACMG classification, primarily due to a lack of available studies or evidence. The class assignment for VUS may change in the future as additional data becomes available, making them unsuitable for inclusion in the initial model creation.

In response to the reviewer's feedback, these explanations have been incorporated into the revised manuscript, along with the expansion of all relevant abbreviations to enhance clarity.

**(Q4) lines 106 – 113: The parameters could be presented in a table.**

**Response:** We appreciate the reviewer's suggestion, and in response, have added the following table to the manuscript:

| **Hyperparameter** | **Value** | **Hyperparameter** | **Value** | **Hyperparameter** | **Value** |
| --- | --- | --- | --- | --- | --- |
| base_score | 0.5 | gpu_id | -1 | min_child_weight | 1 |
| booster | gbtree | grow_policy | depthwise' | missing | nan |
| callbacks | None | importance_type | None | monotone_constraints | () |
| colsample_bylevel | 1 | interaction_constraints | ' | n_estimators | 50 |
| colsample_bynode | 1 | learning_rate | 0.25 | n_jobs | 0 |
| colsample_bytree | 0.9 | max_bin | 256 | num_parallel_tree | 1 |
| early_stopping_rounds | None | max_cat_to_onehot | 4 | predictor | auto |
| enable_categorical | FALSE | max_delta_step | 0 | random_state | 0 |
| eval_metric | None | max_depth | 6 | reg_alpha | 0 |
| gamma | 0.2 | max_leaves | 0 | reg_lambda | 1 |

Reviewer #2: Summary:
Vatsyayan et al. applied two ML models (TabNet and XGBoost) to classify ATP7B genetic variants of Wilsen disease based on highly engineered features of each variant. Both models show very high classification accuracy, indicating the potential usability to reduce the manual evaluation efforts such as following guidelines of American College of Medical Genetics and Genomics and the Association of Molecular Pathologists. However, because of the lack of comparison with other variants classification methods e.g. disease agnostic model, it is hard to tell the novelty of WilsonGenAI and whether WilsonGenAI really adds value to Wilson's disease specific variant classification. Due to the high requirement of storage size of the WilsonGenAI, I have not evaluate the software itself. Please see the following comments for major revision:

Major:
(Q**1). In introduction, please review and discuss related works. Line 174-180 should be part of the introduction.**

**Response:** We appreciate the reviewer's feedback, and in response have added to the introduction section of the manuscript. Specifically, we emphasize that our models are unique in being trained on a gold-standard dataset of variants that have been manually classified according to the ACMG guidelines. Given the diverse underlying disease mechanisms in complex genetic disorders, our models are tailored specifically to Wilson's disease variants.

To the best of our knowledge, there is no existing model trained on a manually ACMG-classified dataset dedicated exclusively to Wilson's disease variants. This distinctive aspect is now highlighted in the revised manuscript, providing a clearer context for the novelty and value of WilsonGenAI in the realm of Wilson's disease variant classification.

**Q2. In results, in addition to CADD, please compare the WilsonGenAI results with more state-of-the-art methods such as Eigen-PC, REVEL, AphaMissense, etc. Moreover, the argument of not comparing the proposed methods with RENOVO and MLVar are not convincing. Please also include these results in Table S3. Without seeing these baseline results, it is difficult to conclude TabNet and XGBoost are necessary Wilson's Disease specific model. The model comparison figure (e.g. barplot of Table S3) might be the main figure highlighted by this paper.**

**Response:** We appreciate the reviewer's valuable suggestion, and in response, we have expanded our model comparisons to include scores from 13 tools: CADD, RENOVO, AlphaMissense, REVEL, SIFT, Polyphen2, Eigen-PC, LRT, MutationTaster, FATHMM, PROVEAN, MetaLR, and MutationAssessor. The results of these comparisons have been incorporated into Supplementary Table 3.

Upon comprehensive evaluation, our models, TabNet and XGBoost, consistently exhibit superior performance across all four metrics. It is noteworthy that XGBoost is outperformed by other models only in Positive Predictive Value (PPV), primarily due to the limited number of variants for which scores were available for other models, which tended to be pathogenic.

In response to the reviewer's suggestion, we have created a barplot that compares all four metrics across the 15 models, providing a comprehensive visual representation of the performance comparison. This barplot, highlighted as Figure S8, has been included in the revised manuscript to better illustrate and emphasize the superiority of TabNet and XGBoost in Wilson's disease variant classification.

**Fig 1.** : Barplot comparing the Accuracy, MCC, NPV and PPV of 13 models with TabNet and XGBoost. Abbreviations: MAssessor - MutationAssessor; MTaster - MutationTaster.

**Q3. line 68, there are much more pathogenic variants than benign class. Have the authors considered whether the imbalanced distribution will influence the results?**

**Response:** We appreciate the reviewer's observation regarding class imbalance. In addressing this concern, for the XGBoost model, we utilized the scale_pos_weight parameter, which adjusts for class imbalance, and is derived by dividing the number of entries in the majority class by the number of entries in the minority class. Subsequently, we employed the mean of cross_val_score function (with CV=10) to assess model performance. The results indicated that the XGBoost model performed optimally with the added weights (MCC = 0.95) compared to when weights were not applied (MCC = 0.90).

Regarding the TabNet model, we addressed class imbalance by setting the weights parameter to '1', enabling automated sampling with inverse class occurrences. While the MCC did not exhibit a change on the training set, the model with weights (MCC = 1) demonstrated superior performance compared to the model without weights (MCC = 0.97) on the test dataset.

To provide a more detailed understanding of the chosen parameters, we have expanded our explanation in the manuscript.

**Q4. line 74-76, it seems the three population used for annotation is different from the population of WilsonGen dataset. Can the authors discuss more on the potential problem of this inconsistency?**

**Response:** We thank the reviewer for raising this concern. The WilsonGen database, which forms the foundation of our study, has meticulously gathered and classified variants from a wide range of sources, including literature and nine comprehensive datasets containing Wilson's Disease mutations. This dataset, to the best of our knowledge, stands as the largest and most comprehensive collection of ACMG/AMP classified variants, incorporating diverse populations globally. Therefore, we expect the models to be able to generalize over variants from different populations.

In our approach, we deliberately opted for allele frequencies from a diverse set of global population datasets during the annotation process, aiming to avoid hyperlocalizing the models to specific population groups. By training the models on the Wilsongen database variants, and annotating them with allele frequencies from these three large and globally representative datasets, we intended to ensure that our models could adequately address the genetic diversity present in different populations. We have expanded on this explanation in the manuscript to provide a clearer understanding of our approach.

**Q5. Figure S1. Can the authors show both training and validation loss in order to easily see whether the model is overfitting or not.**

**Response:** We appreciate the reviewer's suggestion, and in response, we have included a plot with both training and validation losses for the TabNet model. In generating the validation loss, we used a custom cross-entropy loss function, as the TabNet model does not inherently provide validation loss. In the plot below, the original training loss is denoted as 'Loss', while 'Loss_Train' and 'Loss_Val' represent the outputs of the custom cross-entropy loss function. The plot illustrates that both the training and validation losses exhibit a similar trend, with the validation loss slightly higher than the training loss. This alignment suggests a favorable scenario, indicating a lack of overfitting or underfitting. This observation aligns with consistently high Matthews Correlation Coefficient (MCC) scores, reflecting accurate predictions across both classes. Moreover, the model demonstrated perfect accuracy over both classes in the independent test dataset.

**Fig 1.** : Figure depicting the comparison between the train and validation losses

**Q6. Figure S2. It seems the important features identied by XGBoost and TabNet are quite different but their ROC are similar. Can the authors discuss more about this?**

**Response:** We appreciate the reviewer's insightful observation. Both the XGBoost and TabNet models were trained on a comprehensive set of 73 attributes, encompassing various aspects such as positional information of the variant, its prevalence in global populations, pathogenicity prediction scores from different tools, and evolutionary conservation scores. While some of the important features identified by XGBoost and TabNet are distinct, there are important commonalities that highlight shared considerations.

Both models prioritize 10 common features, focusing on Loss of Function (LoF) information, genomic position of the mutation (Start: nucleotide), global prevalence of variants (1000Genomes AF -ALL), as well as pathogenicity scores obtained from 7 prediction tools (MetaSVM, MCAP, MutPred, SIF4G, REVEL, PolyPhen2 HDIV, and MutationTaster). These shared features underscore their own significance in determining pathogenicity.

In addition to the common features, each model incorporates 7 unique attributes each. The XGBoost model considers exonic function, allele frequencies from the GnomAD database, and conservation scores, among others. On the other hand, the TabNet model includes variant prevalence in GnomAD and specific populations, along with additional pathogenicity and conservation scores.

While there are differences in the specific features, it is crucial to note that these models adopt a well-rounded approach by considering various aspects that collectively contribute to variant pathogenicity. The varying features cover similar information, ensuring a comprehensive understanding of the variants.

Furthermore, the training dataset labels are determined through ACMG classification, which accounts for diverse aspects of relevant biological data, including functional and segregational evidence. Consequently, both models capture nuanced patterns among the attributes that contribute to the ACMG classifications, reinforcing the reliability of their predictions.

This nuanced and comprehensive consideration of variant attributes enhances the robustness of both models in capturing the intricacies of variant pathogenicity, as reflected in their similar ROC curves despite differences in important features.

**Q7. Figure S3. It seems the accuracies are very unstable. Can the authors comment on this problem?**

**Response:** We appreciate the reviewer's attention to this aspect. The observed fluctuations in accuracy plots, particularly in deep learning models, are a common occurrence. These fluctuations are often attributed to the training data being fed to the model in batches, a practice employed for computational efficiency. As the model iteratively processes these batches during training, the loss typically decreases, and the accuracy should progressively increase, indicating learning. In the case of our models, the loss and accuracy plots exhibit the expected behavior, with decreasing loss and increasing accuracy over time. While the accuracy may show short-term fluctuations due to batch-wise updates, the overall trend aligns with the model's learning progress.

Q**8. Figrue S4. It is weird that the total number of variants are different between the methods.**

**Response:** The observed difference in the total number of variants between the methods in Figure S4 can be attributed to the specific approach taken during the train-test split process. Both models achieved their best accuracy when applied to a 70-30% train-test split of the 723 variant dataset. However, it's important to note that the TabNet model further divided the 30% test split into equal 50% test and 50% validation subsets.

Consequently, when presenting the confusion matrix, the counts reflect the classification results for the TabNet model on the 50% test subset, leading to 110 classified variants. In contrast, the XGBoost model is represented with the entire 30% test split, resulting in 217 classified variants.

To enhance clarity on this specific aspect, we have included a more detailed explanation in the revised manuscript, ensuring that the distinction in the train-test split methodology is clearly communicated.

**Q9. Since the proposed models only consider two classes, in practice, for the variants with around 0.5 predicted probability, should the user regard them as VUS? Is there a recommended threshold? This is especially important as the authors claimed WilsonGenAI could be used for clinical diagnosis.**

**Response:** We appreciate the reviewer's insightful consideration. In the context of our models, which have learned patterns from ACMG-classified datasets, the predicted probability signifies the likelihood of a variant being either pathogenic or benign based on the presented data. To make a binary decision, we have set 0.5 as the cutoff threshold.

However, recognizing the importance of providing more context, especially for variants with probabilities around 0.5, we have incorporated class probabilities for all VUS variants in Supplementary Table S4. This addition aims to offer users in clinical and research settings a more detailed understanding of the confidence associated with each prediction, facilitating nuanced interpretations, particularly for variants near the 0.5 probability threshold.

**Q10. For the VUS of independent datasets (line 124), is the predicted score of them around the margin of the two classes? Is there a trend or correlation between the predicted score and the 5 ordinal classes?**

**Response:** Out of the 261 Variants of Uncertain Significance (VUS), 251 were exonic and subsequently classified by our models. Among these, 41 variants for TabNet (with a range of probabilities between 0.5000003 and 0.59852874) and 11 for XGBoost (ranging from 0.5096124 to 0.5969398) had predicted probabilities falling within the 0.5 to 0.6 range for either class. Notably, a substantial majority—83.67% for TabNet and 95.62% for XGBoost—had prediction probabilities exceeding 0.60.

To visualize the distribution and range of class probabilities for these variants, the scatterplots below illustrate the probability ranges for all 251 variants for (A) TabNet and (B) XGBoost.

(B)

**Fig 1.** : Scatterplot of class probability vs the predicted class for (A) the TabNet and (B) the XGBoost model across all VUS variants 251 exonic VUS variants that were a part of the validation dataset.

This information provides a comprehensive overview of the predicted probabilities for the VUS variants. The scatterplots convey the distribution patterns and highlight the dominance of variants with probabilities above 0.60, contributing to a more nuanced understanding of the model predictions for VUS variants.

We trained our models using binary classification labels for variants. This is because ACMG sub-classifies variants even within the 5 cardinal classes. Additionally, the final classification may change upon the availability of new information regarding a variant, such as statistical or functional studiesGiven these considerations, we did not anticipate a strong correlation between the binary predictions of our models and the 5 ordinal classes of ACMG.

To offer insight into the class probabilities assigned by our models for the ACMG classes of each variant in the independent set of 156 variants, we present Figures (A) for TabNet and (B) for XGBoost. These figures provide a visual representation of the distribution of class probabilities, emphasizing the model's predictions across the ACMG ordinal classes.

(A)

(B)

**Fig 2.** : Scatterplot of class probability vs the actual ACMG class for (A) the TabNet and (B) the XGBoost model across validation set of 156 variants.

All plots have been added to the manuscript (S6, S7, S9, S10 Figs) as well.

**Q11. Line 189-200, is there a specific consideration to choose S855 and C271X for validation? Ideally, it would be very interesting to see if some VUS with very high predicted pathogenic probability can be validated to lead to low Copper concentration.**

**Response:** We extend our gratitude to the reviewer for highlighting this aspect. The selection of S855F and C271X for validation was driven by a specific research objective related to investigating the functional consequences of Wilson's disease variants. In this context, we chose two variants for comparison: S855F, a variant classified as a Variant of Uncertain Significance (VUS) at the beginning of our investigation, and C271X, a well-established pathogenic variant serving as a positive control for pathogenicity. Additionally, wild-type ATP7B was included as a standard control.

The focus on S855F stemmed from our broader project aimed at functionally validating Wilson's disease variants, particularly those classified as VUS. Through a comprehensive exploration of its functional aspects, we successfully reclassified the S855F variant as pathogenic. This outcome underscores the dynamic nature of variant classifications and the potential for reevaluation based on additional functional insights.

Moreover, our efforts extend beyond these specific variants, as we are actively engaged in functionally validating other similar variants within the same research endeavor. The goal is to uncover the functional implications of various variants, especially those designated as VUS, contributing to a more accurate and nuanced understanding of their pathogenicity.

The importance of investigating VUS lies in the fact that these variants may lead to significant phenotypic consequences, as also highlighted by the reviewer. Causative variants might not have been extensively studied or mapped across diverse populations, and the underrepresentation of certain populations, such as Asians, in large datasets can contribute to the classification of variants as VUS. This underscores the need for ongoing functional and ML-based efforts to unravel the complexities associated with variant pathogenicity.

We appreciate the reviewer's interest in this critical aspect of our research, and we are committed to advancing our understanding of Wilson's disease variants through a multifaceted approach that integrates functional validation and machine learning methodologies.

**Q12. It seems the independent dataset is not available.**

**Response:** The set of 156 variants used as validation set were not a part of the initial WilsonGen database, and were thus not a part of our training dataset of 723 variants. These were collected from previously unreported literature, and only added to the database at a later stage. A lack of any other ACMG-classified datasets of ATP7B variants that could be used as a validation set encouraged us to create one of our own.

Minor:
**Q1. Please define abbreviation WD, VUS before using it.**

**Response:** We appreciate the reviewer's feedback and have incorporated the recommended revisions into the revised manuscript

**2. line 149. Please round the number.**

**Response:** We have prepared the revised manuscript with the suggested modification.
